# Advanced structural and compositional profiling of mineral trioxide aggregate incorporated with nano-carbonated hydroxyapatite: a comprehensive X-ray diffraction and energy dispersive X-ray investigation

**DOI:** 10.2340/biid.v12.45065

**Published:** 2025-12-17

**Authors:** Njwan Fadhel Shehab, Nadia Hameed Hasan, Alaa Edrees Dawood, Nawal Atiya Khalaf

**Affiliations:** Department of Conservative Dentistry, College of Dentistry, University of Mosul, Mosul, Iraq

**Keywords:** Mineral Trioxide Aggregate, nano-carbonated hydroxyapatite, hydration behavior, ATR-FTIR, X-ray diffraction, microstructure

## Abstract

**Introduction:**

The current research tested the assumption that the addition of nano-carbonated hydroxyapatite (nCHAp) to Mineral Trioxide Aggregate (MTA) Angelus would modify its physicochemical properties and alter compositional characteristics that were relevant to the development of bioactive phases, without altering normal hydration dynamics.

**Methods:**

MTA angelus was blended with 5 wt% nano-CHAp and subjected to controlled hydration. Attenuated Total Reflectance Fourier Transform Infrared Spectroscopy (ATR-FTIR) was conducted to identify functional groups and affirm the inclusion of carbonate and phosphate ions. Phase composition was analyzed using X-ray diffraction (XRD). Surface morphology and elemental composition were analyzed by using Field Emission Scanning Electron Microscopy (FESEM) in conjunction with Energy Dispersive X-ray Spectroscopy (EDX). Porosity and microstructural integrity were also examined.

**Results:**

ATR-FTIR spectra showed peaks corresponding to characteristic functional groups of carbonates (CO_3_^2-^) and phosphate (PO_4_^3-^), affirming the incorporation of nano-CHAp chemically. XRD patterns affirmed the preservation of key hydration phases such as portlandite, tricalcium silicate, calcite, and bismuth oxide, with new calcium phosphate phases due to inclusion of nano-CHAp. FESEM images showed more dense microstructure with reduced porosity and reformed particle packing. EDX analysis showed the inclusion of phosphorus and notable increases in calcium and carbon content, corresponding with nano-CHAp inclusion. The modified MTA angelus preserves primary hydration pathways while having enhanced biofunctional availability of ions and surface morphology.

**Conclusion:**

Introduction of nano carbonated hydroxyapatite (nCHAp) brought about discernible changes in the composition of the cement matrix through the phase specific characteristics of XRD, ATR-FTIR, and energy-dispersive X-ray spectroscopy (EDS). These structural changes may create compositional environments that would support the development of mineral-related phases, but it would require further, more specific studies than the current one to confirm biological or clinical outcomes.

## Introduction

Mineral Trioxide Aggregate (MTA) is a calcium silicate-based cement commonly utilized in endodontic and restoration dentistry owing to its superior sealing properties, and biocompatibility. Tricalcium silicate (Ca₃SiO₅) and dicalcium silicate (Ca₂SiO₄), which are the main constituents of MTA, are hydrated to form calcium silicate hydrate (C–S–H) gel and calcium hydroxide (Ca(OH)₂), thus bestowing on it antimicrobial and hard tissue-forming properties. In spite of such favorable properties, MTA has some disadvantages, like delayed setting time, discoloration potential, and unevenness in mechanical strength [1]. There has been increasing interest in modifying MTA in order to enhance its physicochemical and biological characteristics. A potential method to do this is by the addition of nano-carbonated hydroxyapatite (nano-CHAp), which is a bioactive material of structure that is similar to the mineral form of natural dentin and bone. Nano CHAp is a form of hydroxyapatite (HA) that has its phosphate or hydroxyl sites in the crystal lattice replaced in part by carbonate ions; the change has been shown to have an effect on solubility and phase reactivity. When the material is incorporated into calcium silicate cements, such as MTA, the compositional change can precondition the formation of phases that can be associated with bioactive surface properties, which can potentially affect the mechanical properties of the material [2]. There has been increasing interest in the last few years in modifying MTA formulas to better match their physicochemical and biological characteristics. In this study, the nano-CHAp was added not to substitute or outperform the conventional MTA functionality, but as a phase-modification controlling variable to understand the effect the carbonate-containing apatite has in the formation of phases and structural assembly in the MTA framework. The analysis was only limited on compositional and microstructural analysis using X-ray diffraction (XRD), Attenuated Total Reflectance Fourier infrared spectroscopy (ATR-FTIR), and energy dispersive X ray spectroscopy (EDX), without extending to clinical applicability or dissolution-causer behavior.

Carbonated hydroxyapatite (CHAp) is a derivative of HA with the substitution of ions of carbonates (CO₃^2-^) into the lattice of the former. This substitution enhances its resemblance to biological apatite in dental tissues and thus optimizes resorption. The potential of CHAp in dental application, especially in pulp tissue regeneration, has been seen in more recent research based on mineral induction properties and cytocompatibility profiles [3]. The addition of nano-CHAp to MTA formulations can create compositional states that could support the formation of mineral-related phases and could be used to create more densely located areas of mineral deposition on the surface. These structural alterations are apt to influence the material interaction at the interface which can, in its turn, affect the mechanical or sealing behavior; however, these effects should be confirmed by another verification that is not the focus of the current compositional study. The literature on material science has indicated carbonate replacement in nano-CHAp which has a resultant impact on solubility characteristics [4]; however, in the framework of the current research it is treated merely as a compositional variable in the MTA structure. Its presence is studied solely in a structural and phase-interaction perspective and based on the results of ATR-FTIR, (XRD) and energy-dispersive X-ray spectroscopy (EDX) and without further extension of the interpretation to biological or regenerative consequences. The structural and compositional modifications introduced by nano-CHAp addition to MTA are important in assessing its potential in the clinical setting. A key analytical method in this regard is XRD, which is highly efficient in identifying crystalline phases, tracking hydration reactions, and identifying second mineral formation in the cement matrix [1]. Complementing this is the use of energy Dispersive X-ray (EDX) spectroscopy, which provides useful elemental analysis and mapping to evaluate the chemical homogeneity and distribution of major elements [5]. Hence, in this study, the aim is to conduct an enhanced structural and compositional analysis of nano-CHAp modified MTA with the application of XRD and EDX. Through the explanation of crystalline phases and elemental profiles, this research endeavored to explore the hydration mechanisms of the material, along with associated increases in potential impacts of nano-CHAp addition, with implications in endodontic use. The term bioactivity is interpreted to mean in the context of this work the structural detection of the carbonate- and phosphate-related phases that can be an indicator of early ion-interactive potential at the compositional level, as seen through the ATR-FTIR, XRD and EDX. This definition does not suggest ion release behavior, cell responses, or any biological activity in vivo as those were not examined in the current study. This description does not suggest that there is ion-release behavior, cellular responses or any in vivo biological activity since such investigations were not conducted in the current study. The results of this investigation can help towards the establishment of the next generation of commercially available bioactive cements with enhanced clinical properties in endodontic and restorative use.

## Materials and methods

### Materials and sample preparation

Commercial-grade white MTA angelus (Angelus, Londrina, PR, Brazil) was utilized as the base cement. Its major constituents according to the manufacturer’s description are tricalcium silicate (Ca₃SiO₅), dicalcium silicate (Ca₂SiO₄), tricalcium aluminate (Ca_3_Al_2_O_6_), and bismuth oxide (Bi₂O₃) as the radiopacifier. Custom-synthesis of nano carbonated hydroxyapatite (nano-CHAp) powder (99.9% purity; particle size of approximately ~70 nm) was supplied by Nano Research Elements Company (India). To determine the actual morphology and size, the particles were characterized by using FESEM. The FESEM characterization showed that the nanoparticles had a size distribution in the range of approximately 20–70 nm. The modified cement composition (MTAA) was created by mixing with nano-CHAp in amounts of 5% by weight in relation to the total weight of the powders. Accurate weights were provided from the use of a precision digital balance (±0.0001 g). MTA powder content was minimized to provide space to add the nano-CHAp, with the total mass kept constant. The material was mixed by hand in a sterile 5-ml glass beaker under sterile conditions. The nano-CHAp was added gradually to MTA angelus and mixed well until it became homogeneous. The mix was portioned into equal amounts and placed within sterile containers before it was subjected to vortexing on a shaker (as was presented by Hussein, 2019) [6] for 5 s to distribute evenly.

## Hydration protocol and experimental design

MTA Angelus powder was prepared using the recommended ratio of 3:1 of powder and liquid as outlined by the manufacturer. A homogeneous cement paste was prepared by adding about 0.10 g of powder (per calibrated scoop) to 0.03 ml of sterile distilled water using a micro-pipette and mixing them for 30 s using a stainless steel spatula on a sterile glass slab under aseptic condition. In the case of specimen fabrication, the proportionally scaled mass of past was packed into cylindrical molds (10 mm diameter × 2 mm height). The filled molds were subsequently placed in a humidified chamber with a relative humidity of 95% and a temperature of 37ºC to enable a setting period of 24 h. After setting, the samples were ground to fine powder form using agate mortar and pestle. Similarly, prepared unmodified MTA angelus powders were used as control.

## Fourier transform attenuated total reflectance infrared spectroscopy

ATR-FTIR analysis was performed to detect functional groups and validate chemical bonding within the modified material. Analysis of the samples was carried out using an ATR-FTIR spectrometer (ATR-FTIR, Alpha II Bruker, Germany). Spectra were recorded between 400 and 4,000 cm^-1^ with the resolution of 4 cm^-1^ and were mean-averaged from 32 scans. Peaks corresponding to functional groups were observed in both modified and unmodified samples. ATR-FTIR was utilized to check the incorporation of nano-CHAp and to observe changes in hydration products.

## Analysis of X-ray diffraction

Identification of the crystalline phase was performed on a PANalytical X’Pert Pro diffractometer (Phillips Xpert PANanalytical, Holland) with Cu-Kα rays (λ = 1.5406 Å), under an operating voltage of 40 kV and 30 mA. Data were recorded between the range of 2θ = 10° and 80° with scanning speed of 8°/min, and step of 0.05° with 1-s dwell time. The samples were lightly packed into the sample pan with the help of a sterile glass slide to provide surface homogeneity. Identification of the phases was performed by High Score software with JCPDS-ICDD files. Independent phase analyses were performed for nano-CHAp powder, unmodified MTA, and the modified MTAA [7].

## Energy dispersive X-ray spectroscopy in conjunction with field emission scanning electron microscopy

The Field Emission Scanning Electron Microscopy (FESEM) is employed as a descriptive analysis to analyze the surface morphology, form, and particle size. That is accompanied by an EDX employed in both qualitative and quantitative analyses to carry out elemental analysis. Elemental analysis was conducted with the help of the TESCAN MIRA3 FE-SEM system (France) provided with the EDX detector (Oxford Instruments, UK). To sensitize the samples to conductivity, before analysis, the samples were sputter-coated with a gold layer of thickness 20 nm and mounted in the carbon adhesive stub to be tested under FESEM. Five specimens from each group were put under analysis. The operating voltage and the operating current were maintained as 15 kV and 10 mA, respectively.

EDX spectra from three randomly chosen areas from the specimen were recorded with the help of the INCA software (Version 18, Inca Oxford Instruments, Abingdon, UK). Elemental mapping and quantitative point analyses were employed to assess the content of Ca, Si, P, Bi, and C in the CHAp powder and set material (before and after addition of nano CHAp) specimens.

## Results

### ATR-FTIR analysis

**Figure 1:**
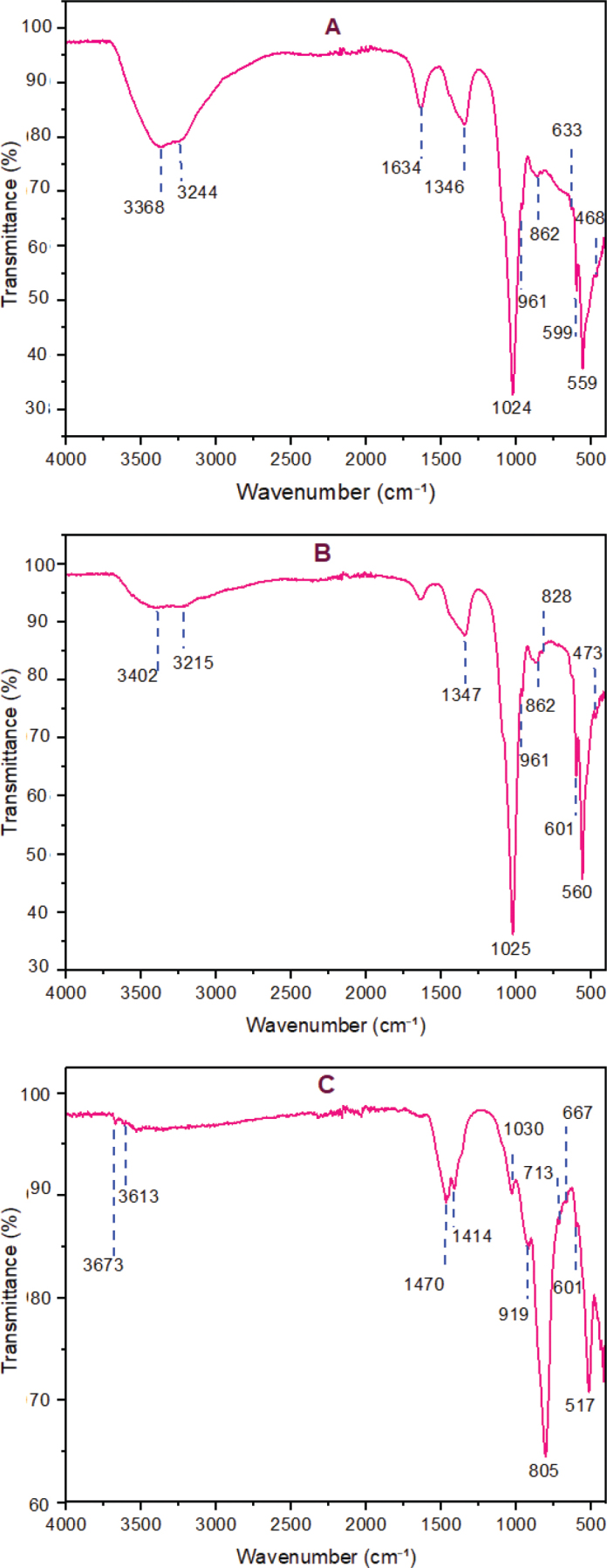
ATR-FTIR spectra of (A) nano-CHAp powder, (B) hydrated MTA Angelus, and (C) MTA Angelus with nano-CHAp modification. Major functional groups are Si–O, PO_4_^3-^, CO_3_^2-^, and O–H vibrations. Rising intensity of the carbonate and phosphate bands in (C) suggests chemical integration of CHA into MTA.

The ATR-FTIR spectrum of nano-CHAp powder included phosphate peaks at 1,024, 961, 599, 559, and 468 cm^-1^; carbonate signals at 1,346 and 862 cm^-1^; and O–H bands at 3,368 and 3,244 cm^-1^ (Figure 1A). Hydrated MTA angelus displayed characteristic vibrational bands at 3,402 cm^-1^and 3,215 cm^-1^, associated with O–H stretching, and a band at 1,635 cm^-1^ indicating H–O–H bending. Peaks at 1,025, 961, 560, and 473 cm^-1^ were attributed to Si–O stretching vibrations, consistent with calcium silicate hydrate. Carbonate absorptions were detected at 1,347 and 862 cm^-1^. Additional low-frequency bands at 828 and 601 cm^-1^ were assigned to unhydrated silicate phases (Figure 1B). In the spectrum of nano-CHAp-modified MTA, new O–H stretching signals appeared at 3,673, 3,613, and 3,533 cm^-1^. Peaks corresponding to phosphate groups were evident at 667 and 601 cm^-1^. Intensified carbonate bands were observed at 1,470, 1,455, and 1,414 cm^-1^. Silicate features at 1,030 and 919 cm^-1^ persisted, with additional bands at 805, 517, and 439 cm^-1^ (Figure 1C) and (Table 1).

**Table 1:** Major infrared bands (in cm–¹) detected using ATR-FTIR spectroscopy.

Sample	O–H Stretch	Si–O Stretch	CO₃²^–^ Bands	PO₄³^–^ Bands
MTA	3402, 3215	1025, 961	1347, 862	–
Modified MTA	3673, 3613	1030, 919	1470, 1414	667, 601
Nano-CHAp	3368, 3244	–	1346, 862	1024, 961

Assignments reflect primary functional groups found in hydrated MTA angelus, nano-CHAp, and nano-CHAp–modified MTA. Bands represent the hydroxyl (O–H), silicate (Si–O), carbonate (CO_3_^2-^), and phosphate PO_4_^3-^) regions.

### Analysis of X-ray diffraction

**Figure 2:**
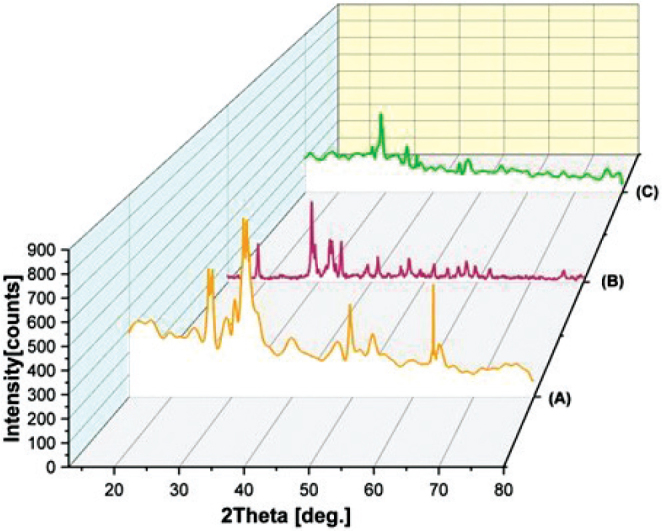
Representative (XRD) patterns of (A) nano-carbonated hydroxyapatite (nano-CHAp), prominent reflections represent crystalline B-type CHAp (B) set MTA Angelus, and (C) MTA Angelus modified with nano-CHAp. Prominent reflections represent crystalline portlandite, bismuth oxide, and calcium silicate phases. Additional peaks in (C) represent calcium phosphate hydrate.

XRD of (nano-CHAp) showed crystalline carbonated HA with no detectable by-products. Diffraction peak with the highest intensity was found to be at 2θ = 32.05° corresponding to (112) crystal plan. Other reflections at 25.72° (002) and 26.87° (210) corresponding to relative intensities of 56.36% and 50.54%, respectively, were found. Peak broadening in (112) corresponding to 32.05° and (004) corresponding to 52.68° reflections with FWHM of 0.5904° and 0.7200° was found. Presence of peaks corresponding to (202), (213), and (222) planes also confirms the single-phase of CHAp with diffraction pattern corresponding to PDF #00-019-0272 (Figure 2A, Table 2). In MTA Angelus set, portlandite was found by reflections corresponding to 2θ = 18.16°, 34.19°, 47.34°, and 50.88° corresponding to PDF #01-076-0570. Bismuth oxide was found in the form of reflections corresponding to 23.06°, 32.84°, and 47.12° and peaks corresponding to tricalcium silicate and dicalcium silicate between 18° and 34°. Reflections corresponding to 29.17° and 39.37° confirm the calcite (Figure 2B). A detailed list of the assignments of phases is tabulated in Table 3. In nano-CHAp containing modified MTA Angelus, diffraction profile consisted of the already seen phases with additional reflections corresponding to 14.68°, 21.33°, 29.03°, 29.59°, 34.47°, and 37.24° corresponding to calcium phosphate hydrate (PDF #00-041-0483). Calcite peaks were more intense, and increased intensity of 34.19° portlandite peak was found (Figure 2C, Table 4).

**Table 2:** Summary of the X-ray diffraction (XRD) peak features of nano-CHAp.

2θ (°)	d-spacing (Å)	FWHM (°)	Intensity (%)	Plane
25.72	3.417	0.2952	56.36	(002)
26.87	3.319	0.1476	50.54	(210)
32.05	2.793	0.5904	100.00	(112)
34.26	2.617	0.3936	36.52	(202)
47.07	1.941	0.5904	16.97	(222)
49.52	1.841	0.3936	28.44	(213)
52.68	1.715	0.7200	24.63	(004)

The data consisted of the peak position (2θ), interplanar spacing (d-spacing), full width at half maximum (FWHM), relative intensity, and the assigned crystal planes. The peaks were indexed based on ICDD-PDF card #00-019-0272.

**Table 3:** Representative crystalline phases observed in set MTA angelus using XRD.

Phase	Reference code	Representative 2θ Peaks (°)
Portlandite	01-076-0570	18.16°, 34.19°, 47.34°, 50.88°, 54.47°
Bismuth Oxide	01-076-2478	23.06°, 32.84°, 47.12°, 50.17°
Tricalcium Silicate	00-014-0693	18.04°, 23.76°, 24.04**°,** 27.27°, 29.35°, 31.53°, 33.05°
Dicalcium Silicate	00-024-0037	18.07°, 23.22°, 27.49°, 29.27°, 31.04°, 32.01°, 34.33°, 51.84°
Calcite	01-086-2341	23.01 °, 29.17 °, 39.37 °, 43.21 °, 46.84 **°,** 57.17 °

Each phase is presented with its characteristic diffraction peaks (2θ) as well as corresponding ICDD-PDF reference codes.

**Table 4:** Additional crystal phases detected in nano-CHAp–modified Angelus MTA.

Phase	Reference code	New Peaks (2θ °)
Calcium Phosphate Hydrate	00-041-0483	14.68°, 21.33°, 29.03°, 29.59°, 34.47°, 37.24°

Peaks match calcium phosphate hydrate phases absent in the unmodified material. Values correlate with PDF #00-041-0483. CHA: Carbonated hydroxyapatite.

### FESEM-EDX analysis

**Figure 3:**
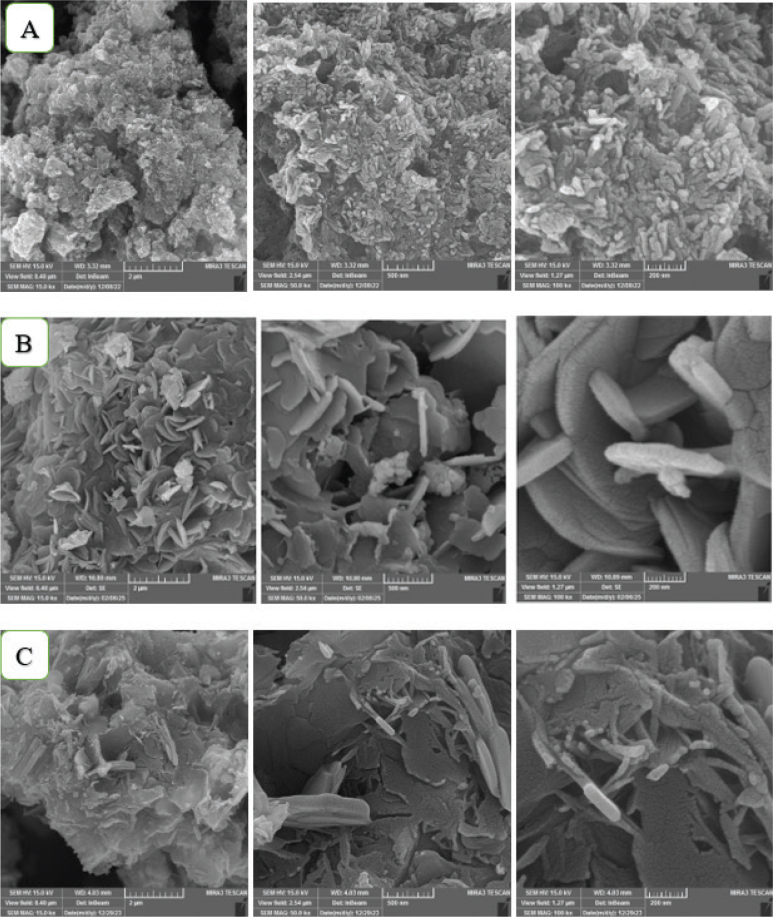
FESEM images of (A) nano-CHAp, (B) unmodified MTA Angelus, and (C) MTA Angelus with nano-CHAp modification. (A) presents porous flaky nanostructures of CHA. (B) reveals open textured and coarse cement particles. (C) presents less porosity of the surface and finer particle integration upon modification.

**Figure 4:**
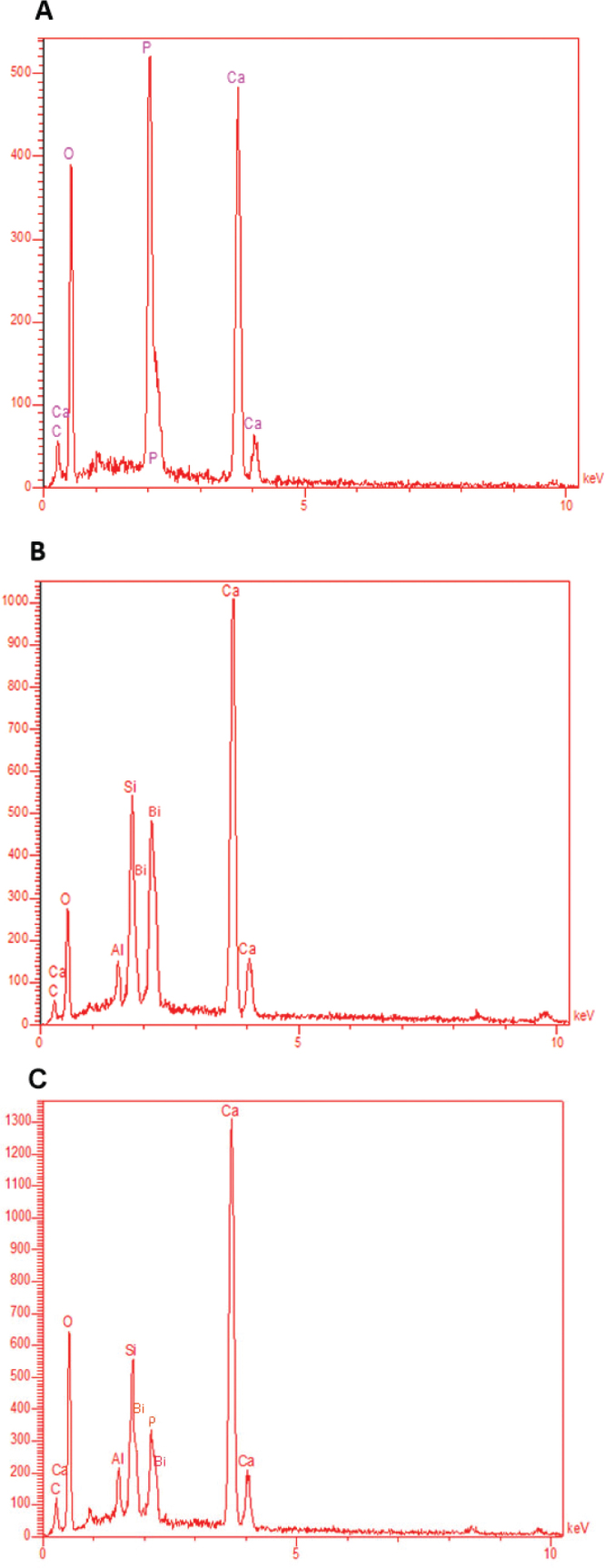
EDX spectra of (A) nano-CHAp, (B) unmodified Angelus MTA, and (C) Angelus MTA modified with nano-CHAp. (A) exhibits elemental composition of B-type CHA with predominant Ca, P, O, and C peaks. (B) exhibits Ca, Si, Bi, Al, and O peaks in agreement with the formulation of MTA. (C) exhibits additional P and higher C peaks, which suggest successful integration of the CHA phase.

Nano-CHAp FESEM micrographs showed flake-like nanostructures and a porous platelet morphology in the submicron range (Figure 3A). Unmodified MTA Angelus demonstrated coarse, angular particles with irregular morphology and open spaces between them (Figure 3B). Modified cement matrix revealed decreased surface porosity and denser grain orientation, with small spherical structures between the MTA particles (Figure 3C). EDX verified the corresponding elemental signature of nano-CHAp with predominant peaks of Ca, P, and O, with detectable levels of C (Figure 4A, Table 5). There were no extraneous elements. In unmodified MTA Angelus, peaks appeared in Ca, Si, Bi, O, and trace C (Figure 4B, Table 6). The modified MTA revealed an EDX signature of the combined elements along with some new P peak and increased Ca and C intensity, with evidence of nano-CHAp addition (Figure 4C, Table 7).

**Table 5:** Energy-dispersive X-ray emission energies and relative intensities, representing elemental peaks for calcium (Ca), phosphorus (P), oxygen (O), and carbon (C), in nano-carbonated hydroxyapatite.

Element	Energy (keV)	Weight%	Intensity
O	~0.5	47.57	High
C	~0.3	9.66	Moderate
Ca	~3.7, ~4.0	24.80	High
P	~2.0	17.97	Very High

**Table 6:** Unmodified MTA angelus elements composition as determined by EDX analysis.

Element	Energy (keV)	Weight%	Intensity
Ca	~3.7, 4.0	35.24	High
Si	~1.75	12.14	Moderate
Bi	~2.4, 2.6	8.31	High
O	~0.5	33.54	Moderate
Al	~1.5	1.93	Low
C	~0.3	8.84	Low

The table is presented with characteristic X-ray emission energies as well as qualitative categories of intensity.

Bi: Bismuth; Si: Silicon; Al: Aluminum.

**Table 7:** EDX elemental profile of nano-carbonated hydroxyapatite modified MTA.

Element	Energy (keV)	Weight%	Intensity
Ca	~3.7, 4.0	36.45	Higher
P	~2.0	2.37	Low
C	~0.3	9.15	slightly higher
Al and Si	As above	1.08 9.32	low and Moderate
Bi	~2.4, 2.6	6.51	slightly lower

There is an additional phosphorus with strengthened carbon peaks as compared to unmodified MTA. Information is indicative of composite composition of the cementitious as well as the apatite phases.

## Discussion

The addition of nano-CHAp into Angelus MTA was examined to analyze its effect on phase composition, microstructure, and elemental distribution – factors that impact the material’s biological and clinical performance in endodontic procedures. Research on the alteration of MTA has shown that the addition of nanoscale additives can influence not only the structure of the material but also the key functional characteristics, such as compressive resistance, ionic dissolution, and antimicrobial effect, which has been reported in previous works on modified formulations of MTA [8–10]. In line with the available literature, compositional and phase-related evidence is presented as part of the current study that forms the basis of further evaluation of these functional consequences under the framework of clinically-focused testing models. ATR-FTIR analysis in this study delivers direct insight into chemical interactions, hydration behavior, and evolution of structure of nano-CHAp modified MTA. The results answer the study’s quest to determine if nano-CHAp addition boosts MTA’s bioactive and physicochemical performance as an endodontic cement. The spectra of the nonmodified MTA revealed broad O–H stretching peaks and characteristic Si–O and Ca–O vibrations confirming the production of calcium silicate hydrate (C–S–H) and portlandite as signs of successful calcium silicate-based cement hydration [11, 12]. The phases of hydration identified using ATR-FTIR coincide with other phases typically reported in the setting progression of calcium silicate-based cements. These stages have been intentionally limited in the current research to the identification and no further to mechanical or sealing performance evaluation as these parameters were not directly measured. The relationship between these stage advancements and functional characteristics, such as compressive strength and sealing ability, is one of the relevant subjects of future directed research. The relative ease of these spectral peaks and decreased resolution indicate a moderate level of crystalline ordering in the set matrix. Conversely, the modified MTA by nano-CHAp had a significantly more complex and differentiated ATR-FTIR spectrum. The narrower and stronger O–H bands at 3,673, 3,613, and 3,533 cm^-1^ – contrasting with the broadened bands in nonmodified MTA – reflect increased portlandite crystallinity and possibly faster or fuller hydration. This result confirms the postulation that nano-CHAp, because of the high surface reactivity and biological imitation of this nanoparticle, can serve as a nucleation site, favoring organized or structured hydration and matrix formation. Carbonate-related absorptions in the modified MTA were also considerably more extensive and stronger with peaks at 1,470, 1,455, 1,414, and 713 cm^-1^. These peaks correspond to B-type carbonate substitution in HA as well as to crystalline calcite formation and evidence double carbonate incorporation mechanisms. The peak at 1,470 cm^-1^ also coincides with B-type carbonate substitution of the HA, where phosphate groups (PO_4_^3-^) are replaced with carbonate ions (CO_3_^2-^) in the structure of the apatite. A B-type substitution of this sort is also found to enhance biological affinity of the HA and is a feature of biologically derived apatites [13]. The strong bands at 1,455 cm^-1^ and at 713 cm^-1^ are characteristic of crystalline calcite (CaCO₃). Their simultaneous presence is a commonly established indicator of calcite [14]. These bands confirm that some carbonation did take place, likely through reaction of environmental CO₂ and calcium-rich hydration products like Ca (OH)₂. Additional indication is provided by the presence of a band at about 1,414 cm^-1^, commonly linked with the formation of carbonate through exposure to environmental CO₂ [15]. The concurrent presence of environmental markers of carbonation confirms that nano-CHAp integrates structurally but also modifies the chemical microenvironment of the setting cement. The observation of carbonate-related phases using the ATR-FTIR is observed in the framework of compositional context of this study. Materials science literature has discussed the phenomenon of carbonate substitution in terms of phase organization and surface precipitation in calcium silicate based cements [13, 16]. In the current research this observation is viewed in the context of a structural property. Any correlation with long-time stability or functional capacity would necessitate special mechanical or ageing assessments and is hence seen as a guide towards subsequent research instead of an end point to the present information. Significantly, the altered material also showed phosphate bend modes at 667 and 601 cm^-1^ missing in the natural sample. Previous materials studies have been linked to compositional conditions that are potentially conducive to mineral phase formation by incorporation of CHA phases into cement matrix. Although these stages include calcium and phosphate elements which are pertinent to mineral interaction, the implication of cellular response at the dentin interface is out of the scope of the present study and would need specific biological analysis [17]. The carbonate and phosphate phases of the diagnostic peaks represent the compositional inclusion of nano-CHAp in the cement structure as indicated in ATR-FTIR and XRD analysis results. This observation is explained with the limitation of spectral analysis and it implies that these phases can be structurally accommodated without the evident disturbance of the matrix. Functional integration would only be confirmed with specific mechanical or biological correlational studies in the future research [10, 14].

The silicate-bounded ATR-FTIR bands at 1,030 and 919 cm^-1^ were retained in the modified formulation, and the new low frequency bands at 517, 476, and 439 cm^-1^ had increased intensity as compared to that of the unmodified MTA [13, 18]. This spectral pattern is indicative of a higher level of arrangement of the calcium silicate hydrate related phases in the matrix [11, 13, 19]. Those observations can only be interpreted in the framework of spectral and phase analysis alone, suggesting a more extensive microstructural arrangement instead of a perturbation of the cementitious web. Although this kind of structural organization is pertinent in terms of materials-engineering viewpoint, especially in terms of controlled phase development, this observation is not reported as direct demonstration of either mechanical reinforcing or clinical sealing behavior since these qualities were not measured in this experiment and would need special-purpose functional testing.

The nano-CHAp was confirmed by XRD to be a B-type carbonate-substituted HA by the presence of the characteristic peaks at 25.72° (002), 26.87° (210), 32.05° (112), 34.26° (202) as observed in Table 2, matching PDF #00-019-0272 and as reported by findings in Torres-Mansilla et al. [20]. The broadness of peaks in the (002) and (210) and (004) reflections indicate the formation of nanocrystalline B-type CHAp in accordance with disorder in the structure brought about by carbonate substitution for phosphate groups – a commonly reported property of biological apatites [2, 21]. Furthermore, the broad full width at half maximum (FWHM), predominantly resolved in the high (112) reflection at 32.05°, is another indication of the existence of nanocrystalline or structural disorder. Such characteristics are very predictable in CHAp materials that have a propensity to form ill-crystalline apatites [22]. The lack of calcite peaks in nano-CHAp also indicates the carbonate as being structurally integrated into the lattice rather than being in the form of free CaCO₃, in accordance with Su et al. [21]. B-type CHAp has been shown to increase osteoclast-mediated resorption, enhance solubility under physiological conditions, and increase osteoconductivity when incorporated into dental materials or bone grafts [23].

The XRD of hydrated MTA angelus modified with nano-CHAp. Figure 3 indicated the occurrence of various crystalline phases, each contributing to the performance and behavior of the material. New peaks at 14.68°, 21.33°, 29.03°, 29.59°, 34.47°, and 37.24° 2θ indicate the formation of a hydrate of calcium phosphate. This phase is only found to be detected in the nano-CHAp -modified MTA, according to characteristic peaks per PDF 00-041-0483. Appearance of this phase is a very strong indication that a reaction has occurred involving MTA components and nano-CHA, likely producing calcium phosphate-rich phases based on the phosphate- and calcium-bearing environment generated by nano-CHAp. Inclusion of nano-CHAp incorporates phosphate ions that favor the origin of phosphate-containing hydration products. These have the potential to increase the bioactivity of the cement to favor the attainment of HA and enhance the biocompatibility of the material [24]. A notable hydration product discovered was portlandite (Ca(OH)₂) with peaks at 2θ values of 18.16°, 34.19°, 47.34°, 50.88°, and 54.47° corresponding to the standard reference PDF #01-076-0570 [25]. These are more intense in nano-CHAp -modified MTA A, and this may reflect higher crystallization of portlandite with nano-CHAp contributing nucleation sites. Higher hydration activity may also be suggested by higher portlandite. Bismuth oxide (Bi₂O₃) was also identified, with peaks at distinct positions of 23.06°, 32.84°, 47.12°, and 50.17°, corresponding to PDF #01-076-2478. This phase is securely incorporated into the structure of MTA to act as a radiopacifier so that the material is viewable under radiographic inspection. Its retention as a crystalline phase indicates that this phase is not involved in the hydration reaction but is chemically stable within the matrix [26]. In the nano-CHAp -modified MTA A, however, no clear change of peak position and/or intensity is observed, suggesting that the radiopacifier is not modified by the nano-CHAp modification. The presence of (Ca₃SiO₅) and (Ca₂SiO₄) was confirmed by a series of peaks that intersect within 18°–51° 2θ, with prominent peaks at 18.04°, 23.76°, 27.27°, 29.35°, 31.53°, 32.31°, 33.05°, and 34.04° corresponding to Ca₃SiO₅ (PDF #00-014-0693), and at 18.07°, 23.22°, 27.49°, 29.27°, 31.04°, 32.01°, 34.33°, and 51.84° corresponding to Ca₂SiO₄ (PDF #00-024-0037) [27]. These unhydrated residues indicate lack of complete reaction, a common phenomenon of hydraulic cements under conditions such as particle size, limited waters available, or slow reaction rates. They however offer a potential long-term store of hydration, contributing to strengthening of the material’s mechanical integrity with time [28]. In the nano-CHAp-altered MTA A, these peaks persist, indicating that alite (Ca₃SiO₅) still lingers but potentially undergoes hydration. No significant peak shift is observed, indicating the same crystallographic structure, but relative intensity is observed slightly decreased, indicative of continuing hydration or dilution with addition of nano-CHAp. However for (Ca₂SiO₄) such peaks are reduced or absent in nano-CHAp -altered MTA, indicative of a probable reaction or conversion of this phase upon hydration with carbonated HA.

Additionally, the diffraction pattern revealed the formation of calcite (CaCO₃), with peaks located at 23.01°, 29.17°, 39.37°, 43.21°, 46.84°, and 57.17° (PDF #01-086-2341) in the set MTA, but more distinctive in the nano-CHAp-modified MTA, with sharper peaks at 29.17°, 36.05°, 39.37°,64.36°, and 76.48˚ (PDF 01-086-2341). This suggests enhanced carbonation, likely from the reaction of Ca(OH)₂ with CO₂, possibly facilitated by the carbonate ions in nano-CHAp, resulting in the formation of calcite as a secondary phase. The formation of calcite is attributed to the carbonation of portlandite upon exposure to atmospheric CO₂ during hydration. Over time, this secondary reaction is common and supports hydration reaction pathways in calcium silicate-based cements that can influence surface characteristics, reduce porosity, and slightly modify mechanical performance [29]. Notably, calcium silicate hydrate (C-S-H), the principal binding phase responsible for the strength and dimensional stability of MTA, was not detected by XRD due to its inherently amorphous to nanocrystalline nature [30].

The XRD graph of hydrated MTA angelus with nano-CHAp modification, Figure 3 reveals the presence of multiple crystalline phases, each conferring performance and behavior to the material. Novel peaks at 14.68°, 21.33°, 29.03°, 29.59°, 34.47°, and 37.24° 2θ reveal the presence of calcium phosphate hydrate. This phase is seen only for nano-CHAp-modified MTA, based on characteristic peaks per PDF 00-041-0483. Appearance of this phase is a testament to a reaction occurring between MTA constituents and nano-CHA, mainly generating calcium phosphate-rich phases, given nano-CHA’s phosphate- and calcium-bearing nature. Inclusion of nano-CHAp introduces phosphate ions to induce phosphate-containing hydration products. These have a potential to enhance the bioactivity of the cement to assist development of HA and increase the material’s compatibility with biological tissues [24]. With the addition of nano-CHAp into MTA, there were added peaks on the XRD corresponding to calcium phosphate hydrate at 14.68°, 21.33°, and 29.03° based on PDF #00-041-0483. This modified MTA. The increased intensity of the portlandite peak at 34.19° in the modified MTA may be an effect of nucleation by the CHAp, as indicated by Abu Zeid et al., who observed a similar behavior with bioactive calcium phosphates [13]. The formation of calcite was also enhanced in the modified product, likely because portlandite was carbonated either upon or after setting [29].

FESEM imaging revealed the nano-CHAp had a flake-like morphology at the nanoscale level, as had been previously described in morphologic analysis of CHA by Priyadharshini et al. [3]. The imaging demonstrated clear microstructural variations for unmodified MTA with a loosely associated particle system and open-textured surfaces characteristic of incomplete hydration in calcium silicate cements [31]. By contrast, in the hybrid system, MTA was modified with the introduction of nano-CHAp and had a denser particle configuration and fewer interstitial pores – a result likely to enhance sealing capacity and minimize leakage which is of clinical importance in retrograde fill and perforation repairs [24]. EDX analysis confirmed elemental addition consistent with structural findings. The addition of phosphorus in the modified MTA – lacking in the nonmodified material – further confirms the integration of nano-CHAp. The increased signals for carbon confirm carbonate substitution. Calcium was the predominant element in all samples but presented a higher signal intensity in modified material, possibly owing to the additive effect of the nano-CHAp phase. These elemental signals are in accordance with what was reported by Shehab *et al*., in bioactive cement composites [32]. Significantly, the elemental peaks for silicon were preserved following modification with CHAp, indicating the introduction of nano-apatite will have no disruptive influence on the cement’s core silicate chemistry. Bismuth intensity in modified MTA is a bit less than in the unmodified one. This reduction is relative and probably occurs because of increased total mass of other elements (particularly Ca and P) from the supplemented nano-CHAp as well as potential slight dilution in Bi₂O₃ concentration since Bi has no presence in CHAp. Nevertheless, Bi remains definitely present and points toward radiopacity being maintained but diminished in relative strength. Integrity of bismuth oxide must be maintained for postoperative imaging, while silicon is key toward setting kinetics and long-term development of strength [11, 26]. These results confirm that the addition of nano-CHAp into MTA angelus results in a structurally stable, chemically complex, and possibly bioactive cement. The development of extra calcium phosphate hydrate phases and enhanced microstructure confirms the hypothesis about the potential biological improvement by the CHA-modified MTA. Further studies need to investigate the clinical significance of this formulation in dynamic conditions such as solubility, cytocompatibility, and in vivo response to tissues. The current experiment selectively focuses on the phase structure, chemical integration, and morphologic development of nC-HAp-modified MTA, investigated with the help of the XRD, ATR-FTIR, and EDX methods. Although these parameters may provide preliminary information on the chemistry of the material and its initial structural arrangement, the next verification of the role in mechanical performance (compressive strength), ion-release rate, and antibacterial activity is an issue to be examined.

## Conclusions

Within the scope of this in vitro study, the addition of (nano-CHAp) to MTA angelus changed the material’s physicochemical profile in a direction beneficial to endodontic use. The reformulated product revealed increased structural integration, diminished porosity, and the development of calcium phosphate-enriched phases, as indicated by the results of ATR-FTIR, XRD, FESEM, and EDX tests. These alterations indicate enhanced hydration chemistry and bioactive potential without affecting the basic elemental configuration of the cement. The results justify continued study of the clinical significance of nano-CHAp-modified MTA in its potential applications to vital pulp treatments, root-end fillings, and regenerative treatments.

## Data Availability

The datasets used to base the conclusions of the work presented here are available from the author to whom correspondence should be addressed on request.
